# The Roles of Social Security and Supplemental Security Income as Determinants of Cognitive Function

**DOI:** 10.1093/geroni/igaf122.4414

**Published:** 2025-12-31

**Authors:** Jiaxing Bao, Daniel Kim

**Affiliations:** School of Community Health and Behavioral Sciences, Bouvé College of Health Sciences, Northeastern University, Boston, Massachusetts, United States; School of Community Health and Behavioral Sciences, Bouvé College of Health Sciences, Northeastern University, Boston, Massachusetts, United States

## Abstract

Alzheimer’s disease and related dementias (ADRD) pose a growing public health challenge in the U.S. and understanding protective factors against cognitive decline has become urgent, with over 6 million older Americans currently affected and projected 2025 costs of $781 billion. Financial security has been identified as a potential determinant of cognitive health, yet the longitudinal effects of social security (SS) and supplemental security income (SSI) on cognitive function remain underexplored. Using nationally-representative individual-level panel data from the Health and Retirement Study (2000-2018) and controlling for state and time fixed effects, we examined the associations between SS and SSI benefits with cognitive function among older adults. The SS analysis included 6,649 individuals aged 67 and older with at least 10 years’ work history; the SSI sub-analysis comprised 971 eligible individuals. Cognitive function was measured using the 27-point Telephone Interview for Cognitive Status (TICS) screening instrument. All models controlled for individual- and state-level covariates and time trends. Each additional $1,000 in annual SS benefits was associated with a 0.014-point increase in the TICS score (P < 0.01). Meanwhile, SSI benefits showed inverse and non-significant associations with cognition. Covariates such as age and comorbidities predicted lower cognitive scores, and health insurance coverage showed strong positive associations with cognition. Overall, SS benefits appear to confer modest and significant benefits to cognitive function among older Americans, suggesting protective effects of financial security on cognitive health. These findings support the importance of public sources of retirement income to help maintain cognitive function in late life for Americans.

